# In Vitro Setup for Determination of Nanoparticle-Mediated Magnetic Cell and Drug Accumulation in Tumor Spheroids under Flow Conditions

**DOI:** 10.3390/cancers14235978

**Published:** 2022-12-03

**Authors:** Jessica Behr, Lucas R. Carnell, Rene Stein, Felix Pfister, Bernhard Friedrich, Christian Huber, Stefan Lyer, Julia Band, Eveline Schreiber, Christoph Alexiou, Christina Janko

**Affiliations:** 1 Department of Otorhinolaryngology, Head and Neck Surgery, Section of Experimental Oncology and Nanomedicine (SEON), Else Kröner-Fresenius-Stiftung Professorship, Universitätsklinikum Erlangen, 91054 Erlangen, Germany; 2 Friedrich-Alexander-Universität Erlangen-Nürnberg, 91054 Erlangen, Germany; 3 RNA Biochemistry, University of Bayreuth, 95447 Bayreuth, Germany; 4 Department of Otorhinolaryngology, Head and Neck Surgery, Section of Experimental Oncology and Nanomedicine (SEON), Professorship for AI-Controlled Nanomaterials, Universitätsklinikum Erlangen, 91054 Erlangen, Germany; 5 Institute of Microwave and Photonics, Friedrich-Alexander-Universität Erlangen-Nürnberg, 91058 Erlangen, Germany

**Keywords:** superparamagnetic iron oxide nanoparticles (SPION), magnetic drug targeting (MDT), magnetic cell targeting (MCT)

## Abstract

**Simple Summary:**

Magnetic nanoparticles can render therapeutics or drugs magnetically guidable, which enables their targeted transport to the diseased tissue in the patient`s body. Before its translation into patients, the efficacy of magnetic accumulation must be tested in cell culture systems. To analyze magnetic enrichment under conditions similar to blood flow, we established an experimental in vitro setup with a tumor spheroid placed in a perfused chamber: with this, we showed that it is possible to accumulate cells or chemotherapeutics at the tumor, increasing their therapeutic efficacy.

**Abstract:**

Superparamagnetic iron oxide nanoparticles (SPIONs) are used in nanomedicine as transporter systems for therapeutic cargos, or to magnetize cells to make them magnetically guidable. In cancer treatment, the site-directed delivery of chemotherapeutics or immune effector cells to the tumor can increase the therapeutic efficacy in the target region, and simultaneously reduce toxic side-effects in the rest of the body. To enable the transfer of new methods, such as the nanoparticle-mediated transport from bench to bedside, suitable experimental setups must be developed. In vivo, the SPIONs or SPION-loaded cells must be applied into the blood stream, to finally reach the tumor: consequently, targeting and treatment efficacy should be analyzed under conditions which are as close to in vivo as possible. Here, we established an in vitro method, including tumor spheroids placed in a chamber system under the influence of a magnetic field, and adapted to a peristaltic pump, to mimic the blood flow. This enabled us to analyze the magnetic capture and antitumor effects of magnetically targeted mitoxantrone and immune cells under dynamic conditions. We showed that the magnetic nanoparticle-mediated accumulation increased the anti-tumor effects, and reduced the unspecific distribution of both mitoxantrone and cells. Especially for nanomedical research, investigation of the site-specific targeting of particles, cells or drugs under circulation is important. We conclude that our in vitro setup improves the screening process of nanomedical candidates for cancer treatment.

## 1. Introduction

Cancer is one of the most common causes of death. In 2018, 17 million people were newly diagnosed with cancer worldwide [1]. Depending on the type of cancer, surgery, chemotherapy or radiotherapy are usually performed to achieve a curative remission [2]; nevertheless, only 50% of all cancer patients survived for 10 or more years in 2010/2011 [3]. To improve the prognosis of patients, intensive research is being done in the field of cancer treatment. In 2018, numerous manuscripts were published in the field of cancer research [4]. In nanomedicine, targeted drug delivery is being studied especially, which could positively affect the destruction of tumors, and improve the chances of being cured [5].

The use of drug-loaded superparamagnetic iron oxide nanoparticles (SPIONs) dates back to the 1970s [6]. The inner iron core is magnetic, and the surrounding coat can be changed and customized to achieve a non-toxic, biocompatible nanoparticle, which can be loaded with chemotherapeutics (i.e., mitoxantrone, MTO). After intra-arterial applications, SPIONs can be navigated to the tumor region, using an external magnet, a process which is referred to as “Magnetic Drug Targeting” (MDT). MDT leads to a selectively higher amount of the drug in the tumor, and a reduction of unspecific distribution in healthy tissues, which minimizes side-effects [7,8]. In previous works, we used SPIONs coated with lauric acid (LA) and bovine (BSA), or human serum albumin (HSA) loaded with MTO [9,10,11,12]. These particles effectively killed tumor cells in suspension, in conventional two-dimensional (2D) cell culture, but also were highly effective when applied to three-dimensional (3D) tumor spheroid structures [13,14,15,16,17]; efficacy was also demonstrated in vivo [8]. 

In addition to the targeting of drugs, SPIONs can also be used to magnetize cells by attachment to the plasma membrane and/or uptake to enable their magnetic navigation, referred to as “Magnetic Cell Targeting” (MCT). We demonstrated, previously, that SPIONs are biocompatible, depending on their coating [9,11,18,19]. Citrate-coated SPIONs can be taken up into or attached to T cells, which renders them magnetically guidable [20,21,22,23]. The SPIONs were biocompatible with murine T cell lines and primary human T cells, and the effector functions of the cells were not affected by the SPIONs. We also showed, in vitro, that magnetic accumulation of SPION-loaded T cells is possible [20,21].

Ideally, experiments performed in vitro should predict the outcome in vivo. In the past, several studies performed in standard 2D cell culture could not meet this requirement, as the experimental conditions were too artificial. Multicellular tumor spheroids represent a system of intermediate complexity between monolayer cell culture and in vivo systems [24,25,26,27,28]. Due to cell-cell interactions and formation of extracellular matrices, tumor spheroids develop morphological and functional characteristics comparable to the corresponding in vivo tissue [29]. In spheroids, gradients of oxygen and nutrients, and catabolic products exist, which lead to differences of the cells within the spheroid: proliferating cells at the periphery; non-proliferating cells in the middle; and necrotic cells in the core of the spheroid, analogous to tumors [28]. Thus, spheroids can serve as a tool to investigate drug penetration and efficacy in avascular tumors, such as micrometastases [30]. Spheroids are particularly relevant in nanomedicine research, as penetration of therapeutic nanoparticles in tumor tissues may be limited, due to the presence of extracellular matrices and cell-cell contacts [31,32]. Additionally—and importantly for the investigation of nanoparticle-targeting strategies, magnetically or by other methods (aptamer, antibody, ligand-based)—phenomena such as sedimentation and fluid dynamics of the blood must be independently taken into account.

In order to address forces and conditions under blood flow in the human body, we performed experiments with magnetic nanoparticles ex vivo in human umbilical cord arteries [33,34]. For our first pilot experiments, targeting tumor spheroids under flow conditions, we immobilized colon carcinoma spheroids in channels melted into agarose, which were perfused with a peristaltic pump in the presence or absence of a magnetic field [17]. Although this approach enabled us to investigate the magnetic accumulation of nanoparticles under perfusion, only a few experiments were performed, due to the complicated production and recovery of the spheroids; therefore, we aimed to use a commercially available setup, consisting of transwell chambers carrying spheroids or organoids under flow conditions (MIVO^®^) [35]: in our opinion, the combination of both would be ideally suited to analyzing the magnetic accumulation and the effect of SPIONs or SPION-loaded cells in a standardized setup.

In the present study, we investigated the treatment of tumor spheroids with drug-loaded SPIONs and SPION-loaded immune cells under dynamic conditions in the presence or absence of a magnetic field: we showed that the nanoparticle-mediated accumulation of both—the drug-loaded SPIONs and the SPION-loaded immune cells—increased the anti-tumor effects, and reduced the unspecific distribution of both. We conclude that the MIVO^®^ system can serve as a valuable tool, especially for nanomedical investigations.

## 2. Materials and Methods

### 2.1. Synthesis of SPIONs Carrying Mitoxantrone (SPION^MTO^)

Caffeic acid pentafluoro phenyl ester (CafPFP) was synthesized, following the method of Williams et al. [36]. Details about the synthesis are given in Appendix A. The CafPFP powder was stored dry and with light protection until further use.

Caffeic acid-stabilized SPIONs (Caf-SPIONs) were synthesized by adapting a protocol described by Mühlberger et al. A detailed description of the synthesis procedure can be found in Appendix A [21]. All particle batches were synthesized in triplicate (*n* = 3).

Albumin modification was achieved by slightly changing the protocol of Zaloga et al. to covalently bind albumin to Caf-SPIONs [11]. Briefly, 38 mL of Caf-SPIONs with a total iron content of 100 mg was added to 10 mL of a 20 *w*/*v*% bovine serum albumin (BSA) solution, while stirring at 600 rpm using an overhead stirrer. After 2 min of stirring, 2 mL of 0.1 M NaOH solution was added. The reaction solution was further stirred for 24 h at room temperature. Once the reaction was completed, 50 mL of di. H_2_O was added, to dilute the particle dispersion for washing by tangential flow filtration (KrosFlo Research II, Spectrum Labs, Waltham, MA, USA). The SPIONs were washed from unbound albumin with 500 mL of di. H_2_O, using a 100 kDa hollow fiber filter module, and concentrated back to a total volume of 25 mL. The washed Caf-BSA-SPIONs (in the following, referenced with SPIONs) were stored at 4 °C until further use.

MTO was added directly to the SPIONs with a fixed Fe/MTO weight-ratio of 100, to achieve the following target concentrations of c(MTO) = 5 µM, 0.5 µM or 0.05 µM ± 10%, and c(Fe) = 225 µg/mL, 22.5 µg/mL or 2.25 µg/mL, respectively. The resulting MTO-loaded SPIONs were referred to as SPION^MTO^.

### 2.2. Synthesis of Cit-SPIONs for Immune Cell Loading

The citrate-stabilized SPIONs (Cit-SPION) were synthesized by an adjusted protocol of Elbialy et al. [37], following the steps described in Boosz et al. [20]. Briefly, iron (II) and iron (III) salts were dissolved in H_2_O at a molar ratio of 1:2. After mixing in an inert atmosphere, aqueous ammonia was rapidly injected into the salt solution, to precipitate the SPIONs. After a 15 min growing stage, a sodium citrate solution was added to the SPION dispersion for surface stabilization. The temperature was raised to 90 °C, and the dispersion was mixed for 30 min. After cooling to room temperature, the SPIONs were washed five times using acetone, then dried and redispersed in H_2_O.

### 2.3. Physicochemical Characterization of SPION Systems

Hydrodynamic size (Z-Average), polydispersity index (PDI), zeta potential at pH 7.3, volumetric susceptibility, and iron content characterization of the different SPION systems were measured, following the protocols of Mühlberger et al. [22].

MTO loading onto the SPIONs was measured using the HPLC-UV method of Tietze et al.

Briefly, 460 µL of SPION^MTO^ was centrifuged at 13,000× *g* for 15 min. Then, 100 µL of the particle-free supernatant was diluted 1:10, using 900 µL of H_2_O for HPLC-UV detection. HPLC was performed using a Waters Alliance model consisting of a separation module (2695 series), a dual wavelength absorbance detector (2487 series) and a liquid phase of 20% (*v*/*v*) methanol and 80% (*v*/*v*) 80 mM formate buffer at pH 3 with a flow rate of 1 mL/min and a column temperature of 55 °C. A 3.0 × 100 mm X-Bridge Phenyl column (Waters Corporation, Milford, MA, USA), with a particle diameter of 3.5 μm, was used as the column; 50 µL of samples was injected and analyzed at 254 nm. A calibration curve was measured between MTO concentrations of 250 ng/mL and 5000 ng/mL. As a control, MTO was mixed with H_2_O instead of SPIONs, using the same procedure as described in 2.1. The loading efficacy of MTO onto the SPIONs was calculated as follows:

$$Loading\;efficacy=\left(1-\frac{MTO\;concentration\;in\;supernatant\;of\;SPIONs}{MTO\;concentration\;in\;supernatant\;without\;SPIONs}\right)\times 100\%$$

### 2.4. Cells and Culture Conditions

A375M melanoma cells (ATCC, Manassas, VA, USA) were cultured in Roswell Park Memorial Institute (RPMI) 1640 medium (Thermo Fisher Scientific, Waltham, MA, USA), supplemented with 10% fetal calf serum (FCS, Gibco, Life Technologies Corporation, New York, NY, USA), 1% penicillin/streptomycin, 1% L-Glutamine, 0.04% Beta-Mercapto-Ethanol (all from Thermo Fisher Scientific, Waltham, MA, USA) and 0.2% Hepes Puffer (Biowest, Nuaillé, France).

Fibroblasts were cultivated in Dulbecco’s Modified Eagle medium (DMEM) (Biochrom Cat FG 0045, PAN Biotech, Aidenbach, Germany) with 10% FCS and 1% each of Amphotericin B (Thermo Fisher Scientific, Waltham, MA, USA), penicillin/streptomycin, L-Glutamine and non-essential amino acids (NEAA) (Merck KGaA, Darmstadt, Germany).

Jurkat T lymphocytes (ACC 282, DSMZ, Braunschweig, Germany) were cultivated in RPMI 1640 medium (Thermo Fisher Scientific, Waltham, MA, USA), supplemented by 10% FCS and 1% L-Glutamine.

PBMCs were isolated from peripheral human blood obtained from healthy volunteers after informed consent (approved by the ethics committee of the Friedrich-Alexander-Universität Erlangen-Nürnberg, reference number 257_14 B). For that, blood was drawn in citrate-containing tubes (Monovette SARSTEDT AG & Co. KG, Nürnbrecht, Germany). Next, 8 mL each were placed in a Vacutainer CPT Tube (Becton Dickinson, Franklin Lakes, NJ, USA) that was centrifuged for 20 min at 2900 rpm (5810R, Eppendorf, Hamburg, Germany). The lymphocyte and monocyte layers were collected and pipetted into a pre-warmed 15 mL Falcon, which contained 12 mL of Phosphate Buffered Saline (PBS, Thermo Fisher Scientific, Waltham, MA, USA) and 3 mL of FCS. The cell suspension was washed with 10 mL of PBS, and the cells were collected in RPMI medium containing 10% FCS, 2% penicillin/streptomycin, 2% Amphotericin B, 1% L-Glutamine and IL-7 (Immunotools, Friesoythe, Germany). The PBMCs were counted with a Neubauer counting chamber, using 0.1% Trypan blue (Biowest, Nuaillé, French).

All cells were cultured in a humidified incubator (Memmert, Schwabach, Germany) at 37 °C, 95% humidified air and 5% CO_2_. To analyze the viability and number of cells, a MUSE Cell Analyzer (Merck Millipore, Billerica, MA, USA) was used.

### 2.5. Cultivation of Spheroids

Wells of 96-well plates (TPP, Trasadingen, Switzerland) were each pre-coated with 50 µL of 1.5% Agarose (Carl Roth GmbH + Co KG, Karlsruhe, Germany) in distillated water.

To grow spheroids, A375M cells and fibroblasts were detached by 2.5 mL of 0.05% trypsin (PAN Biotech, Aidenbach, Germany), and counted. 20 µL containing 15 × 10^3^ A375M cells, and 80 µL containing 8 × 10^3^ fibroblasts, were added to130 µL of A375M medium. These 200 µL were seeded into each agarose-pre-coated well of the 96-well plates. The cells were incubated for 72 h, to form spheroids.

### 2.6. Treatment of Spheroids with MTO, SPION^MTO^ and SPIONs under Static Conditions

First, we wanted to analyze if the exposure time of MTO had different effects on spheroid growth. Three days after seeding, the spheroids were treated with MTO (20 mg/10 mL, Hexal AG, Horzkirchen, Germany). MTO was diluted in di. H_2_O, and was added to the spheroids to reach final concentrations of 5.0, 0.5 and 0.05 µM. H_2_O-treated cells served as controls. After 7 days, all the spheroids were harvested.

To compare the effects of SPIONs, MTO or SPION^MTO^ on the spheroids, the spheroids were prepared from 10.5 × 10^3^ A375M cells and 5.6 × 10^3^ fibroblasts in a 96 round bottom well plate (Corning Incorporated, New York, NY, USA), and incubated. On the 3rd day, 50 µL of MTO, SPIONs, SPION^MTO^ or di. H_2_O (ctrl) were added to each well, so that the spheroids received MTO and/or iron amounts as shown in Table 1.

### 2.7. Determination of Spheroid Growth

To determine the growth of the spheroids only treated with MTO, transmission microscopy pictures were taken on days 0, 1 and 4 after the treatment, with an Axiovert 40 CFL Microscope (Carl Zeiss, Oberkochen, Germany) and a 2.5× objective, using AxioVision SE64 Rel4.9 software (Zeiss, Jena, Germany). ImageJ software (National Institutes of Health, Bethesda, Rockville, MD, USA) was used to measure the diameters of each spheroid, horizontally and vertically.

IncuCyte (Sartorius, Göttingen, Germany) was used to analyze the growth of the spheroids treated with SPIONs, MTO or SPION^MTO^ over a total period of 10 days. Data analysis was performed with IncuCyte 2020 B (Sartorius, Göttingen, Germany), ImageJ and MS Excel.

### 2.8. Harvest of the Spheroids, and Analysis in Flow Cytometry

The spheroids were harvested on day 7, after being synthesized. From each condition, 8–9 spheroids were inserted into 1.5 mL Eppendorf tubes, and centrifuged for 3 min at 400× *g* (5430R, Eppendorf, Hamburg, Germany). The supernatants were removed, and the spheroids were washed with 500 µL of PBS. After centrifugation for 3 min at 400× *g*, the supernatant was discarded, and 100 µL of 0.05% trypsin was added and incubated for 6 min. The cells were pipetted up and down to receive single-cell suspensions. 500 µL of A375M medium was added, and the cells were centrifugated for 5 min at 400× *g*. After removing the supernatant, the cells were resuspended in a 50 µL of A375M medium.

The staining solution for the flow cytometry was freshly prepared from 5 mL of Ringer’s solution (Fresenius Kabi, Bad Homburg vor der Höhe, Germany) with 5 µL of Hoechst 33342 (invitrogen by Thermo Fisher Scientific, Carlsbad, CA, USA), 10 µL of AnnexinA5-fluoresceinisothiocyanate (Ax-FITC) (Immunotools, Friesoythe, Germany) and 10 µL of propidium iodide (PI, Sigma Life Science, St. Louis, MI, USA).

250 µL of the mixture was added to cells achieved from 3–4 (*n* = 2, 3) spheroids per condition, incubated for 20 min at 4 °C and analyzed by flow cytometry (Beckman Coulter, Fullerton, CA, USA). Fitc and PI were excited at 488 nm; the Fitc fluorescence was recorded on a Fluorescence1 (FL1) sensor (525/38 nm band pass filter, BP), and the PI fluorescence on an FL3 sensor (620/30 nm BP). Excitation of the MTO fluorescence was at 638 nm and was recorded on an FL7 sensor (725/20 nm BP). Hoechst 33342 was excited at 405 nm and was recorded on an FL9 sensor (430/40 nm BP). Data analysis was performed with Kaluza software Version 2.0 (Beckman Coulter, Fullerton, CA, USA).

### 2.9. Embedding of Spheroids, Preparation of Frozen Sections and Histochemistry

4–8 spheroids were collected, washed, fixed with 4% formalin (Carl Roth GmbH + Co KG, Karlsruhe, Germany) and put into an Eppendorf tube. The tubes were filled up with Tissue-Tek (Sakura Finetek Europe B.V., Alphen aan den Rijn, Netherlands), and were frozen at −20 °C. The next day, frozen sections (thickness of 15–22 µm) were prepared from the spheroids, using a Cryostat (SLEE, Nieder-Olm, Germany), and were stored on microscope slides (Gerhard Menzel GmbH, Braunschweig, Germany): slideswere directly fixed with an aqueous mounting agent for microscopy (Aquatex, Merck KGaA, Darmstadt, Germany), stained with Hoechst 33342, covered and analyzed with the fluorescence microscope.

### 2.10. Investigation of Effect of MTO, SPION^MTO^ and SPIONs on Spheroids under Dynamic Conditions

To investigate the effect of SPION^MTO^ and MTO on spheroids under dynamic conditions, we used a peristaltic pump and MIVO^®^ single flow chambers (both React4Life, Genova, Italy). Six tube systems were used in parallel.

The experimental setup is presented in Figure 3A. The roller of the peristaltic pump circulated and powered the flow in the first tube, I (33 cm), which was connected to a shorter, second tube, II (12 cm), then the chamber followed. The chamber was joined to a third, short tube, III (12 cm), which was linked to a three-way valve linking to tube I again. The chamber was cut to create a shorter distance between magnet and fluid.

First, tube III was filled with 1 mL of A375M medium, using a 3 mL shot (Omnifix, Braun, Melsungen, Germany). Then, 1.5 mL of medium was injected into tube I through the three-way valve. Next, 0.5 mL of compounds (MTO, -SPION^MTO^, SPIONs or di. H_2_O) was inserted into tube I, using a 1 mL syringe (Omnifix), followed by another 0.3 mL of medium (Figure 3A).

Two magnets (Webcraft GmbH, Uster, Switzerland) were placed around the chamber. The disc magnet on the top had an adhesive force of 6.5 kg, while the neodymium magnet on the bottom had a diameter of 15 mm, and an 8 mm height with an adhesive force of 6.2 kg.

The system ran at a speed of 1.08 mL/min for 1 h in the incubator. The purple tube (tube I) was replaced by a new tube (tube I_2_) filled with fresh A375 medium, and at the same time about 12 spheroids (after 3 days of growing) were transferred into each chamber. Samples were taken out of tube I. The pump ran for another ½ h, before tube I_2_ was replaced again with a new tube (tube I_3_) filled with fresh A375M medium. Samples were taken out of tube I_2_. After another ½-1 h of flowing time, samples were taken out of tube I_3_, as well as the chamber, and the spheroids were harvested. All spheroids from one chamber were collected into one well of a 12-well plate. Then, 750 µL of A375M medium was added to each well, and afterwards the spheroids were transferred separately, in a total volume of 100 µL of suspension, into agarose-coated 96-well plates. Their growth was observed for 72 h.

### 2.11. Sample Collection and Determination of MTO Amount in HPLC

The samples were collected from tube I, tube I_2_, tube I_3_ and the chamber, and were measured using Waters e2695 high-performance liquid chromatography (HPLC) (Waters Corporation, Milford, MA, USA); 50 µL per sample (*n* = 2,3) was diluted with 150 µL of di. H_2_O. To identify the concentration, a standard range was determined with every experiment. Data analysis was performed with EmPower (Waters Corporation, Milford, MA, USA) and MS Excel.

### 2.12. Determination of Iron Content from Particles Using Atomic Emission Spectroscopy (AES)

To prove the accumulation of SPION^MTO^ in the chamber with magnets, all the fluids were taken out of the chamber. Next, 2–3 Eppendorf tubes were filled with 20 µL per condition, and were each diluted with 80 µL of nitric acid. The solution was shaken by a ThermoMixer C (Eppendorf, Hamburg, Germany) at 95 °C at 300 rpm for 15 min. The suspension was cooled down, centrifuged for 10 sec and diluted with 1900 µL of H_2_O. The atomic emission spectrometer 4200 MP-AES (AES; Agilent Technologies, Santa Clara, CA, USA) measured the iron content of the samples.

### 2.13. Determination of Iron Content from Cit-SPION-Loaded Jurkat Cells Using AES

2.5 × 10^5^ Jurkat cells were seeded in 12-well plates, and were incubated with 0, 20, 40, 60 or 80 µg/mL of Cit-SPIONs overnight. The cells were transferred into 1.5 mL Eppendorf tubes, and were washed two times with PBS to remove excess particles. The pellets were resuspended in 0.4 mL of PBS, and cells were counted, using the MUSE Cell Analyzer. The cells were sedimented (300 rcf; 5 min; RT), and the pellets were dried at 95 °C for 30 min at 300 rpm. Thereafter, lysis of the pellets was achieved by the addition of 65% nitric acid and a subsequent incubation at 95 °C for 15 min at 300 rpm. Then, the lysed cells were diluted (1:8) with di. H_2_O, and the iron content were measured by AES. Finally, the iron content (pg) per cell was calculated, by dividing the iron amount by the number of cells in the sample.

### 2.14. Loading of Cells with Cit-SPIONs and Magnetic Accumulation

The Jurkat cells were counted with the MUSE Cell Analyzer, and 7 × 10^6^ cells each were put in four culture flasks (TPP, Trasadingen, Switzerland) with a 75 cm^2^ area. Then, 1 mL of 50 µg Fe/mL Cit-SPIONs or di. H_2_O (ctrl) was added, each to two of the four flasks, which were stored overnight in the incubator at 37 °C. The cells were washed three times with PBS or medium, and counted again. Then, 9 × 10^6^ cells per each condition were used.

The MIVO^®^ pump was operated as explained previously. Per condition, 3 mL of Jurkat medium was injected, as well as Jurkat cells or Cit-SPION^Jurkat^ containing 9 × 10^6^ cells resuspended in 400 µL of Jurkat medium.

The flow was set up for 1 h in the presence (+ mag) or absence (− mag) of two magnets around the chamber. The disc magnet on the top had an adhesive force of 6.5 kg; the pot magnet on the bottom had an adhesive force of 30 kg. After 1 h, the fluid in the chamber was collected. The chamber was rinsed with 200 µL of PBS. The cells were counted using a MUSE Cell Analyzer.

### 2.15. Treatment of Spheroids with PBMCs in a Dynamical In Vitro System

The PBMCs were diluted by the RPMI medium, to yield a final concentration of 1 × 10^6^ cells/ mL, and two 75 cm^2^ flasks were filled, each with 15 × 10^6^ cells. They were stimulated with the ImmunoCult™ Human CD3/CD28/CD2 T Cell Activator (StemCell Technologies, Vancouver, Canada), using 25 µL/mL, and were stored in the incubator at 37 °C, 95% humidified air and 5% CO_2_. After 48 h, 200 µL of Cit-SPIONs, with a concentration of 80 µg/mL, was added to one flask and incubated overnight; cells treated with 200 µL of di. H_2_O served as controls.

On the 3rd day after stimulation, the cells were counted again, using the MUSE Cell Analyzer. The pump system was built up, as stated before: 2.5 mL of RPMI medium was injected, as well as 0.5 mL of medium (control) or 0.5 mL of the PBMC solution containing 2.5 × 10^6^ cells; six spheroids were added into each chamber. The flow was set up for 1 h in the presence or absence of the two magnets. The magnetic setup was the same as described in 2.10 (Figure 3A–C). Thereafter, all spheroids and the solution of one chamber were harvested in one well, and supplemented with 700 µL of RPMI medium. Then the spheroids were each embedded with 200 µL into a 96 round bottom well plate (Corning Incorporated, New York, NY, USA), and were observed for 5 days.

### 2.16. Magnetic Field Simulation

The accumulation of SPION-loaded drugs/cells was formed by the application of an external magnetic field from permanent magnets. To determine the magnetic field acting on particles or cells, simulations were performed using COMSOL. The physical relationships exploited and solved in the simulation were the divergence of the magnetic flux density B, and the dependence of B on the magnetic field strength H. As magnetic charge did not exist, the divergence of B was always zero: ∇⋅B=0.

The relationship between B and H was linear, with the permeability $\mu :\;B=\mu H=\mu_{0}\mu_{r}H,$ where $\mu_{0}=4\pi \cdot 10^{-7}$ H/m, and $\mu_{r}$ was the relative permeability; $\mu_{r}$ is a measure of the permeability of a medium relative to that of a vacuum.

Two permanent magnet configurations, as illustrated in Figure A2, were considered for the simulation: the geometric details are shown in Figure A3, and are further described in Table A1.

In the first simulation, a simulation volume (“plate”) with the desired geometric dimensions and magnetic properties, replaced the volume in which the SPION-loaded drugs or cells were present. The thickness of the plate was set to 10 μm, which corresponded approximately to the thickness of the SPION-loaded drugs or cells. The plate was placed parallel above the magnet, to simulate the magnetic field and the corresponding force on the SPION-loaded drugs or cells in the xy-plane. It was assumed that the SPION-loaded drugs or cells were homogenously distributed in this volume.

The SPION suspension has a volumetric magnetic susceptibility of $\chi_{mass}=4.322\times 10^{-3}$ m^3^/kg (measured for 1 mg iron/1 mL). This suspension was mixed with $9\times 10^{6}$ cells. The simulation considered that an average 1 pg of SPION bound itself to the cells. The cells were assumed to be only water and spheres with a radius of 1.5 µm. The volume of a sphere was

$$V_{cell}=\frac{4}{3}\pi r^{3}\approx 14.37{\;\mu m}^{3}≜14.37\;\mathrm{pL}$$

With this volume on the weight of the SPIONs in the cell, we had a ratio of

$$ratio=\frac{1\;\mathrm{pg}\;\mathrm{iron}}{14.37\;\mathrm{pL}}≜\frac{1}{14.37}\frac{1\;\mathrm{mg}}{1\;\mathrm{mL}}.$$

which showed that the volumetric magnetic susceptibility was

$$\chi_{mass,cell}=\frac{1}{14.37}\cdot 4.322\cdot 10^{-3}\frac{m^{3}}{\mathrm{kg}}\approx 0.3\cdot 10^{-3}\frac{m^{3}}{\mathrm{kg}}.$$

For the simulation, the density of the SPION-cell was assumed to be like water, as the ratio between SPION and water/cells is negligible. The relation between the relative magnetic permeability and the volumetric magnetic susceptibility was given by

$$\mu_{r}=\chi_{m}+1=\chi_{mass}\cdot \rho +1,$$

where $\chi_{m}$ was the magnetic susceptibility. With the density of water $\rho_{water}=1000$ kg/m^3^ the relative magnetic permeability was

$$\mu_{r}=\chi_{mass}\cdot \rho_{water}+1=1.3.$$

Afterwards, the cells were mixed with the Jurkat medium, but now it was assumed that the volumetric magnetic susceptibly would not be lowered any more, as the particle cell suspension was assumed to be stable, and we wanted to compute the magnetic field that influenced the SPION-loaded cells. The cells were represented by a simulation volume, with a magnetic relative permeability of about 1.3 in the simulation.

We assumed that the simulation by Caf-BSA-SPIONs loaded with MTO would lead to similar results.

### 2.17. Data Analysis and Statistics

The data were analyzed in MS Excel (Microsoft Corporation, Redmond, WA, USA) and Graphpad Prism 9 (GraphPad Software, San Diego, CA, USA). The pictures were designed with BioRender (BioRender, Toronto, Canada), and were adjusted with PowerPoint (Microsoft) and Adobe Photoshop (Adobe Inc., San Jose, CA, USA).

## 3. Results

### 3.1. Physicochemical Characterization of SPIONs

**Figure A1:**
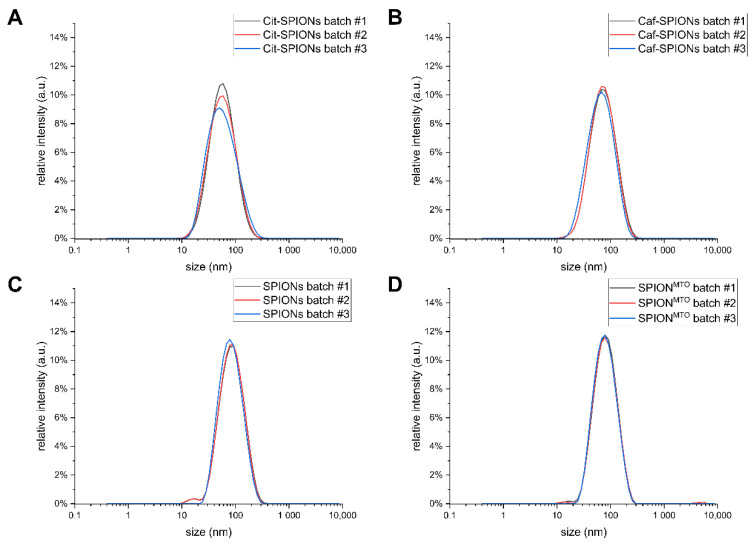
Size distribution of (**A**) Cit-SPIONs, (**B**) Caf-SPIONs, (**C**) SPIONs and (**D**) SPION^MTO^.

**Figure A2:**
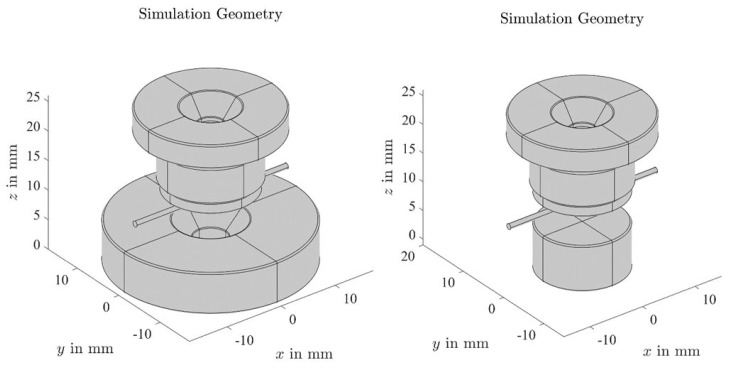
3D geometry of the magnet-SPION configurations.

**Figure A3:**
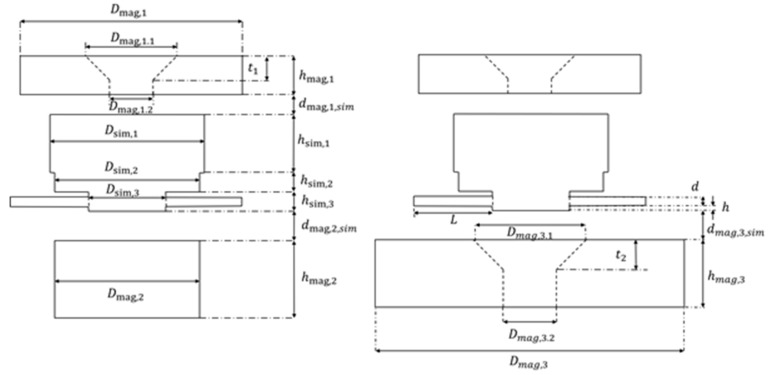
Parameters of the simulated geometries. The setup on the left was used for the enrichment of drug-loaded SPIONs, while the setup on the right was used for SPION-loaded cells.

The SPIONs were physicochemically characterized as summarized in Table 2. The size distributions of the various SPION systems are shown in Figure A1. The Cit-SPIONs for immune cell loading exhibited a hydrodynamic diameter of 51 ± 1 nm, with a corresponding PDI value of 0.208 ± 0.051. The ζ-potential at pH 7.3 was −44.6 ± 9.1 mV. The Cit-SPIONs exhibited a magnetic volumetric susceptibility of 4.32 ± 0.18.

The Caf-SPIONs, which were synthesized using the adapted Cit-SPION synthesis protocol [20,37], exhibited a slightly larger hydrodynamic diameter of 63 ± 4 nm, with a corresponding PDI value of 0.213 ± 0.014. While the ζ-potential at pH 7.3, with a value of −47.7 ± 6.5 mV, was in the same range as that of the Cit-SPIONs, the magnetic volumetric susceptibility was found to have a lower value of 3.36 ± 0.05.

After functionalization of the precursor Caf-SPIONs with BSA, referred to as SPIONs, the hydrodynamic diameter increased to 73 ± 2 nm, with a corresponding PDI value of 0.225 ± 0.011. A decrease to −33.9 ± 4.9 mV in the ζ-potential at pH 7.3 was observed after functionalization, whereas only a slight change of the susceptibility to 3.11 ± 0.12 was found.

After loading the SPIONs with MTO, resulting in SPION^MTO^, the particles showed an unaltered hydrodynamic diameter of 70 ± 1 nm, with a corresponding PDI value of 0.193 ± 0.004. The MTO binding efficiency of the SPIONs was 64% ± 4%: thus, 6.4 ± 0.4 ng MTO was loaded onto 1 µg of iron.

### 3.2. MTO Induces Cell Death in Spheroids in a Time- and Dose-Dependent Manner

**Figure 1:**
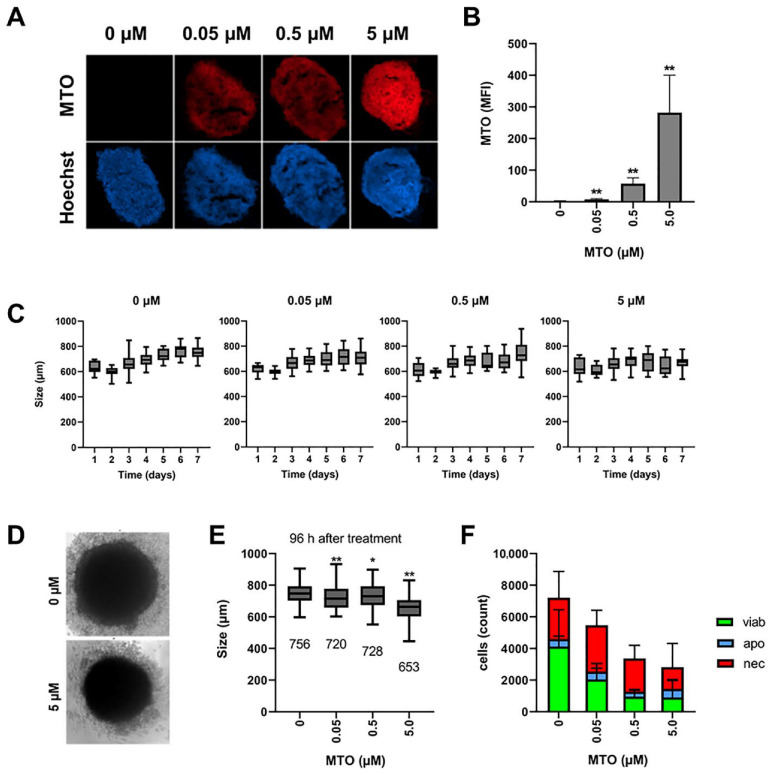
Accumulation of MTO and induction of cell death in spheroids. Spheroids were cultured from A375M cells and fibroblasts in agarose-coated wells for 72 h, then mitoxantrone was added. (**A**) Fluorescence microscopy of cryosections of the spheroids on day 7. (**B**) Single-cell suspensions of the spheroids were analyzed in flow cytometry for intracellular MTO fluorescence on day 7. (**C**) Transmission microscopy and determination of spheroid size by Image J software. (**D**) Transmission microscopy after 96 h of incubation with MTO and (**E**) size determination. (**F**) AxA5-PI staining of single cell-suspensions on day 7. The experiment was performed four times with *n* > 18 (**C**–**E**), or three times in triplicates (**B**,**F**). The mean values with standard deviations are shown. Significances (treatment versus control) were calculated using one-way ANOVA with * *p* ≤ 0.01 and ** *p* ≤ 0.05.

To form spheroids, A375M cells were incubated with fibroblasts for 3 days. Then, 72 h after seeding, the spheroids were treated with 0, 0.05, 0.5 or 5 µM of MTO, to investigate toxicity dependent on the dose and incubation time. Spheroid size was documented in transmission microscopy every 24 h. After 7 days of incubation, the spheroids were harvested for analysis in flow cytometry or for the preparation of cryosections. MTO uptake in cells and spheroids was analyzed based on its inherent fluorescence [38]. With increasing MTO concentration, the spheroids took up more of the cytotoxic drug, and were smaller and less compact, as depicted in the fluorescence microscopy of the frozen spheroid sections (Figure 1A). Increased MTO amounts in the cells were confirmed by flow cytometry, after the dissolution of the spheroids into single-cell suspensions (Figure 1B). When we analyzed the growth of the spheroids over a period of 7 days, by measuring their diameters, we found in all the samples a decrease in spheroid size until day 2, due to the formation of a compact structure. The untreated control spheroids showed typical growth behavior [39]: from day 2 onwards, they constantly increased in size, until they reached a plateau on day 6. With the addition of MTO, the increase in size reduced, and was even abolished by the highest MTO concentration of 5 µM (Figure 1C).

Figure 1D shows pictures of transmission microscopy after 96 h of treatment, clearly indicating differences in size between an untreated and a 5 µM-treated spheroid. With 0.05 and 0.5 µM, the spheroids were significantly smaller than the untreated controls; however, the diameters of both treatment groups were very similar (Figure 1E). When we analyzed single-cell suspensions from the spheroids, we found necrotic cells in the controls as well, which was expected, because of the relatively large size of the spheroids [39]. With longer incubation time and increased size, spheroids have been reported to develop necrotic cores. With increased amounts of MTO, the absolute cell number, and also the number of viable cells, decreased (Figure 1F), confirming the correlation of spheroid size and cell count. In the literature, spheroid size has been used as a simple and fast readout after treatment with cytotoxic drugs [40,41]. Although spheroid size seemed not to be a very sensitive marker, differences between the untreated control and the drug-treated samples were significant: as it is fast, easily measurable, and applicable to semi-automated data analysis, we used it as a marker in our study. The use of a viability marker, such as acid phosphatase, which can be applied to 3D cell culture as well, might improve the data [42].

In sum, we saw the dose-dependent toxicity of MTO. The goal of chemotherapeutic treatment is the maximal killing of the tumor cells. As the dose of MTO to be applied to the patient is limited by its toxicity against healthy tissues and cells, the tumor-directed targeting of the drug would increase its efficacy, and reduce side-effects. To enable the targeting of the drug to the wanted area, MTO was loaded onto the SPIONs, which were magnetically guidable.

**Figure 2:**
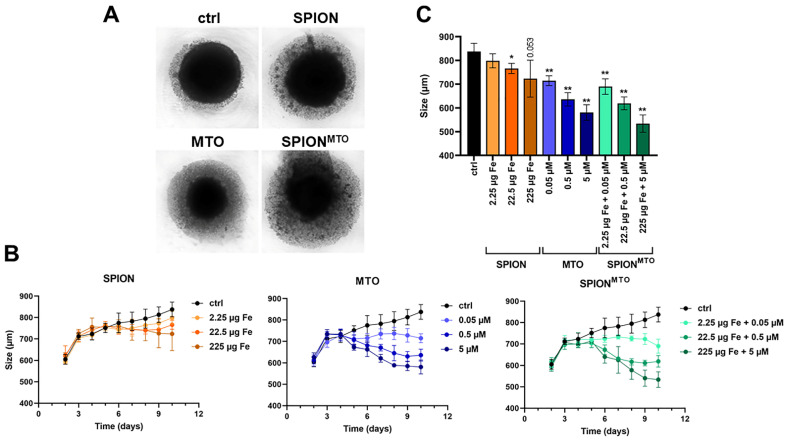
Impact of free and nanoparticle-bound MTO on the growth of spheroids. Spheroids were cultured from A375M and fibroblasts. After 72 h, free MTO, SPION^MTO^ or unloaded SPIONs were added. (**A**) Transmission microscopy of spheroids on day 10. (**B**) The growth of the spheroids was observed with IncuCyte, and analyzed with ImageJ over 10 days. (**C**) Comparison of spheroid sizes on day 10. The experiment was performed in quadruplicates. The mean values with standard deviations are shown. Significances (treatment versus control) were calculated using one-way ANOVA, with * *p* ≤ 0.05, ** *p* ≤ 0.01 (**B**).

For a first comparison of the effects of free MTO, SPION^MTO^ and SPIONs on spheroids, a static experimental setup was chosen. Then, 72 h after seeding, free MTO, SPIONs or SPION^MTO^ were added to the spheroids, together with untreated controls, in a 96-well plate, and their growth was analyzed by live cell microscopy, using the IncuCyte system. Figure 2A shows the spheroids as observed in transmission microscopy on day 10. The untreated controls had a compact inner core, with virtually no detached cells. The SPION-treated spheroids were slightly smaller, with more detached cells. After treatment with MTO or SPION^MTO^, the size of the inner core was reduced, and at the same time the number of detached cells was increased (Figure 2A).

Analyzing the sizes of the spheroids over 10 days, we saw that the free MTO and its nanoparticle-bound counterpart SPION^MTO^ strongly reduced the growth of spheroids in an MTO dose-dependent manner. After 10 days, the untreated controls reached a size of approximately 840 nm, whereas treatment with 5 µM of MTO (free or nanoparticle-bound) resulted in spheroid sizes of approximately 600 and 580 nm, respectively. Although SPION^MTO^ resulted in smaller spheroids, the effect was not significant. Unloaded SPIONs also reduced the growth of spheroids, but to a lesser extent (Figure 2B,C): this may have been due to the formation of ROS caused by the iron surfaces [43].

### 3.3. Magnetic Accumulation of SPION^MTO^ in a Dynamic Flow System

**Figure 3:**
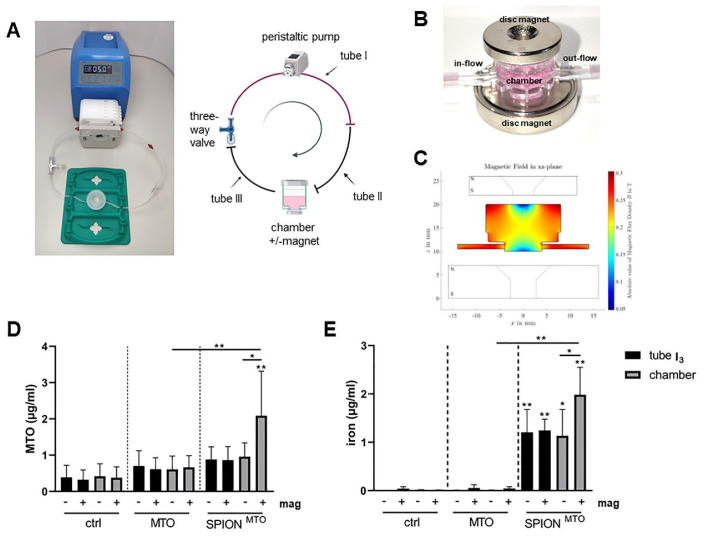
Magnetic accumulation of SPION^MTO^ in a dynamic flow system. Spheroids were incubated in a chamber connected to a peristaltic pump with two tubes. MTO or SPION^MTO^ (MTO amount 2.25 µg) were applied in a bolus. Tube I was exchanged three times with the containing fluid, to mimic distribution of MTO out of the blood circulation into tissues. Samples were analyzed from tube I_3_ and the chamber containing the spheroid, after 2.5 h of running. (**A**–**C**) Experimental setup with overview (**A**), magnetic setup (**B**) and simulation of the magnetic field (**C**). The amount of MTO was determined by HPLC (**D**). 225 µg of SPIONs were applied in bolus, and accumulation in the chamber in the presence or absence of a magnetic field was analyzed using atomic emission spectroscopy (**E**). The experiment was performed three times. The mean values with standard deviations are shown. Significances were calculated within the groups (tubes and channels), using two-way ANOVA: * *p* ≤ 0.05, ** *p* ≤ 0.01.

Ideally, in vitro experiments should be able to predict the in vivo outcome; thus far, we had analyzed the impact of free MTO, SPION^MTO^ and free SPIONs under static conditions. For the treatment of tumors in vivo, this setup was not appropriate, as the drugs were applied not in a static system, but in the blood, which was circulating in the body. To mimic blood flow and the tumor region, we applied a peristaltic pump connected with tubes to a chamber containing a tumor spheroid. MTO, SPION^MTO^ or SPIONs were applied in a bolus. To accumulate the SPIONs in the tumor region, magnets were placed around the chamber containing the spheroid (Figure 3A–C). Altogether, the pump was operated for 2.5–3 h, pumping medium through the system. To simulate the distribution of the applied drug out of the vessels into the tissues, the tubes with the containing medium were exchanged at three time points.

To analyze the nanoparticle-mediated accumulation in the presence and absence of a magnetic field, the amount of MTO as well as the amount of iron in the chamber was determined, using HPLC. We also analyzed the MTO content in the system after each change of the tube: as expected, the amount of MTO in the system decreased with every change of the tube (data not shown). Figure 3D depicts the amount of remaining MTO in the chamber and the tube after the third change. As expected, MTO measurements of the controls only showed background levels. With free MTO applied, more MTO was detected in the tube and the chamber. The application of a magnet did not increase the MTO amount in the chamber. In contrast, with SPION^MTO^, similar levels of MTO were detected in the tube and in the chamber (without magnet), which were also comparable to those measured for free MTO. In the presence of a magnet, however, SPION^MTO^ were accumulated in the chamber (Figure 3D).

When we analyzed the iron content for SPION^MTO^ in the tubes and the chambers, using atomic emission spectroscopy, we found that with magnets significantly more nanoparticles could be enriched in the chamber than without magnets. The amount in tube I_3_ was not altered by the magnet (Figure 3E).

### 3.4. Size of Spheroids after Magnetic Accumulation of SPION^MTO^ in a Dynamic Flow System

**Figure 4:**
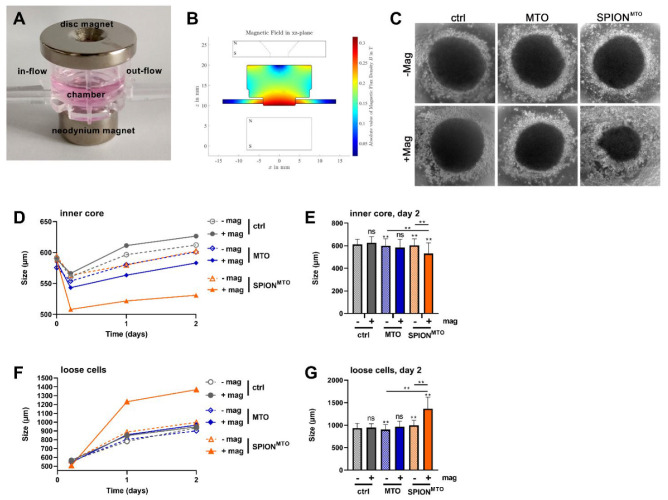
Magnetic accumulation of SPION^MTO^ and effect on spheroids in a dynamic system. Spheroids were cultured for 3 days, and placed in a peristaltic pump connected to a MIVO^®^ chamber with two tubes. Free MTO, SPION^MTO^ or unloaded SPIONs were pumped for 2.5–3 h with 1.08 mL/min in the presence or absence of a magnetic field (**A**). Simulation of the magnetic field in the chamber (**B**). Spheroids were harvested from the chambers, including the surrounding medium, then transferred into agarose-coated 96-well plates, and observed for 48 h in transmission microscopy (**C**). Pictures were analyzed using ImageJ software (**D**–**G**). The size of the compact inner core of the spheroid (**C**,**D**) was evaluated separately from the surrounding detached cells on day 2 (**D**,**E**). The experiment was performed three times, with 4–24 spheroids per condition (**D**–**G**). The mean values with standard deviations are shown. Significances were calculated using the two-way ANOVA test, with ** *p* ≤ 0.01, ns = non-significant (**E**,**G**).

To analyze the impact of MTO or SPION^MTO^ on spheroids in a dynamical flow, the spheroids were transferred, 72 h after seeding, into the chamber of the MIVO^®^ pump system. The setup and procedure were nearly identical to the above-described, except that the bottom magnets were replaced with a neodymium magnet (Figure 4A). After the 2.5 h run, which included three changes of one of the tubes, the spheroids and the surrounding medium were harvested from the chambers, transferred to 96-well plates containing fresh medium and observed for 48 h. Then, the sizes of the compact inner core of the spheroid, as well as the detached cells, were analyzed.

After 48 h, we observed virtually no difference, in regard to the size of the inner core, between the control spheroids and the spheroids treated with MTO, whether with or without a magnet. Furthermore, the size of the detached cell cloud was similar in the control spheroids and the spheroids treated with MTO. Treatment with SPION^MTO^ without a magnet showed the same picture. With magnetic accumulation, spheroids treated with SPION^MTO^ had a smaller inner core and more detached cells, probably representing dying and dead cells (Figure 4C–G); however, for a clear identification of cell death, a live-dead staining must be performed: for instance, calcein AM (green) and propidium iodide (red) had been used by others previously as markers to discriminate viable and dead cells in spheroids in fluorescence microscopy [44].

### 3.5. Accumulation of Jurkat T Cells in a Dynamical System

Thus far, we had magnetically accumulated SPIONs loaded with MTO intended for targeted chemotherapy (Figure 3 and Figure 4). With magnetic accumulation, the tumor spheroid size was reduced by SPION^MTO^ (Figure 4). Here, SPIONs served as a transporter system for the drug.

In the same way, SPIONs can be used to make cells magnetically guidable. We previously loaded T cells with SPIONs, for use in targeted immune therapy [20,21,22,23]. Here, we used the MIVO^®^ system to investigate the magnetic accumulation of SPION-loaded Jurkat T cells under dynamic flow conditions. For the loading of cells, Citrate-coated SPIONs had been developed and characterized previously [21,22].

**Figure 5:**
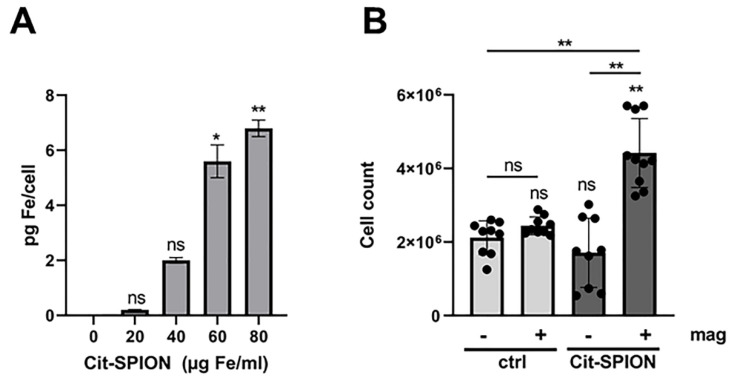
Enrichment of SPION-loaded Jurkat cells in a dynamic pump system. (**A**) Jurkat cells were loaded with Cit-SPIONs overnight. Cellular iron content was measured with AES. (**B**) Cit-SPION-loaded as well as non-loaded Jurkat cells circulated in the dynamic system for 1 h, in the presence or absence of magnets around the chamber. After 1 h, the cell count was determined in the chamber. The experiment was performed once (**A**) or three times in triplicate (**B**). The mean values with standard deviations are shown. Significances were calculated using nonparametric Kruskal-Wallis (**A**) or one-way ANOVA (**B**) with * *p* ≤ 0.05, ** *p* ≤ 0.01, ns = non-significant.

According to previous findings, using primary human T cells and the mouse T cell lines EL4 and BW5147.3, SPIONs associate with Jurkat T cells in a dose-dependent manner [20,21,23]. With 80 µg/mL of Cit-SPIONs, an iron load of up to 7 pg per cell was achieved in Jurkat cells (Figure 5A). Next, we gave the Cit-SPION-loaded Jurkat cells into the MIVO^®^ pump-system. Again, we used a magnet system around the chamber, to accumulate the cells. After 1 h flowing time, we took samples, and determined the number of cells in the chamber. We found approximately 2 × 10^6^ non-loaded control cells in the chambers, no matter if a magnet was present or not. With Cit-SPION-loading, Jurkat cells were enriched when a magnet was present (Figure 5B).

### 3.6. Enrichment and Effect of Stimulated PBMCs

The immune system can elicit powerful anti-tumor immune responses. We used isolated stimulated PBMCs to induce an unspecific anti-tumor response. To analyze the magnetic accumulation of PBMCs and their effect on tumor spheroids, we unspecifically stimulated the PBMCs with CD3/CD28/CD2 for 3 days, and loaded them overnight with Cit-SPIONs. Then, the Cit-SPION-loaded PBMCs were given into the dynamical pump system with or without magnets at the spheroid-containing chambers. The cells were pumped for 1 h and, subsequently, the spheroids and the accumulated PBMCs were isolated from the chamber, and incubated together for a further 5 days.

**Figure 6:**
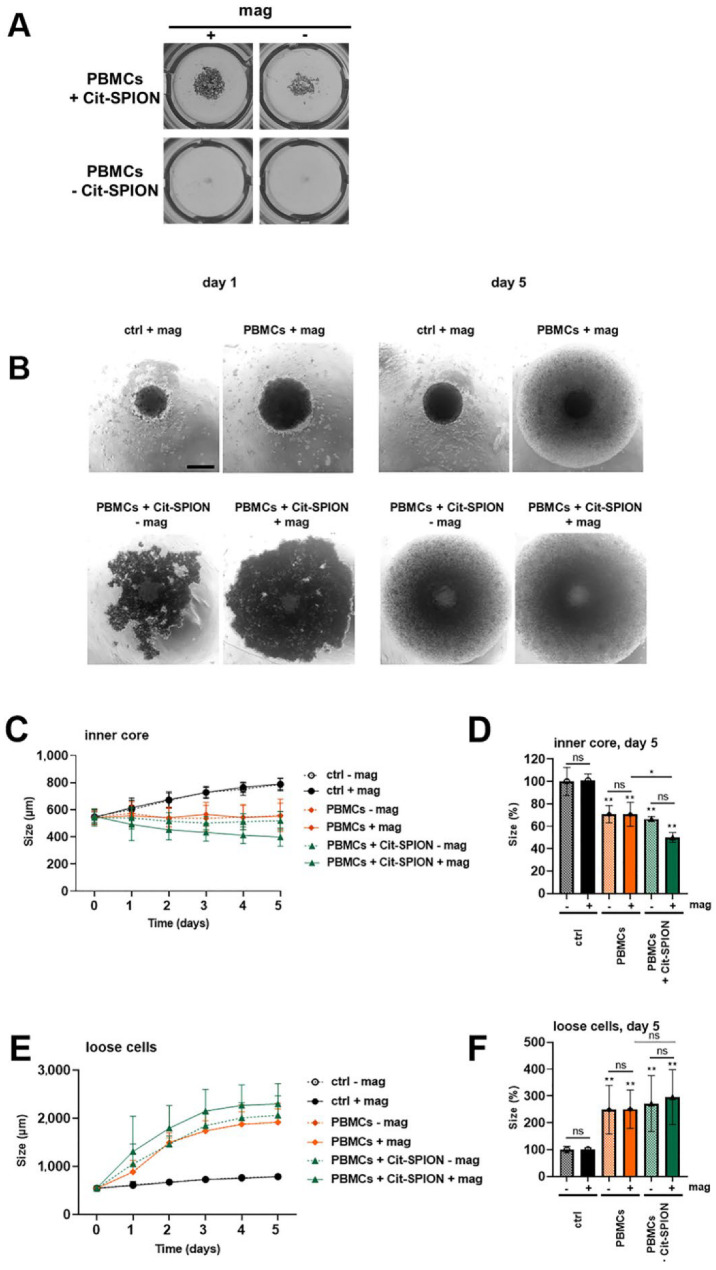
Magnetic accumulation of Cit-SPION with immune cells in a dynamic system. Spheroids were cultured for 3 days, and placed in a peristaltic system connected to a MIVO^®^ chamber. Media, Cit-SPION-loaded or non-loaded PBMCs were pumped for 1 h with 1.08 mL/min in the presence or absence of a magnetic field; afterwards, the spheroids were harvested from the chambers, including the surrounding medium. Macroscopic (**A**) and microscopic (**B**) images of spheroids and enriched PBMCs from the chamber after 1 h of pumping (**A**,**B**) (**left**) or 5 days of incubation (**B**) (**right**). (**C**,**D**) The size of the inner core (compact tumor spheroid) was evaluated separately from the surrounding detached cells (**E**,**F**). The experiment was performed three times. The mean values with standard deviation are shown. Significances were calculated using the two-way ANOVA test with * *p* ≤ 0.05, ** *p* ≤ 0.05, ns = non-significant.

Even macroscopically, we could observe an accumulation of Cit-SPION-loaded PBMCs under flow conditions. SPION-loaded cells were accumulated efficiently with a magnet—but also, interestingly, without a magnet, SPION-loaded cells accumulated more than the unloaded controls, which may have been due to the increased sedimentation of the loaded cells (Figure 6A). Transmission microscopy confirmed this suggestion. Without SPION-loading, only a few PBMCs were found in the chamber together with the spheroid. With SPION-loading, cells accumulated in the chamber, an effect which could be further enhanced in the presence of a magnet (Figure 6B (left)).

We analyzed the size of the spheroids after 5 days of incubation with the accumulated PBMCs. Microscopy pictures of day 5 are depicted in Figure 6B (right). We noticed a significant reduction in the size of the inner compact core when treated with Cit-SPION-loaded PBMCs with magnetic accumulation, compared to the controls (Figure 6B–D). Additionally, we observed and quantified an increase of loose cells around the spheroid, which probably represented proliferating PBMCs, as well as detached tumor cells. With the magnetically enriched Cit-SPION-loaded PBMCs, the increase of these loose cells was most pronounced (Figure 6B,E,F).

## 4. Discussion

The improvement of cancer treatment is still one of the most important topics in research worldwide. New treatments and substances are usually developed and tested in cell culture, which is an artificial approach that often fails to predict the in vivo outcome [45,46,47]. The use of appropriate 3D model systems and in vitro methods considering the circulation in the body may lead to more accurate outcomes than basic 2D cultures in a static setup. This is even more important for the use of nanoparticles, where sedimentation of the latter strongly influences the results [48]. Here, we used melanoma spheroids placed in chambers under constant flow conditions to analyze the effect of magnetic accumulation of drug-loaded SPIONs (Figure 1, Figure 2, Figure 3 and Figure 4) and SPION-loaded PBMCs (Figure 5 and Figure 6).

In our approach, SPIONs served as a magnetically guidable transporter system for drugs or for cells. In vivo, we and others had already proved the efficacy of magnetic targeting to the tumor region [8]. With SPIONs as drug transporters, a depot of the drug could be created by magnetic accumulation, inducting stronger effects on the tumor cells. In contrast to earlier studies by our group, where MTO was attached to non-covalently bound albumin on the SPIONs, in this approach the drug was bound to covalently attached albumin on the SPION surface, to reduce leakage of the drug-albumin complex from the nanoparticles. We previously showed in suspension cells (Jurkat T cells) and adherent monolayer culture (HT-29 colon carcinoma cells) that MTO loaded onto SPIONs induced the same cell death phenotype as its soluble counterpart, but that the velocity of cell death induction was slightly delayed [13,16]: this may have been due to a slower cellular uptake of the particles, compared to soluble drugs. In line with these results, we showed in 3D cell culture, using HT-29 spheroids, that SPION^MTO^ initially accumulated on the spheroid surface before penetrating [15]. In this static setup, however, it was not possible for us to investigate the effect of magnetic enrichment.

Ideally, in vivo effects should be predicted by in vitro experiments: thus, we aimed to establish a system which enabled the investigation of the anti-tumor efficacy of magnetically accumulated particles or immune cells against spheroids. Hitherto, others had performed investigations on tumor spheroids with nanoparticles under flow conditions, but without magnetic fields [49,50]. We had already performed a pilot study employing colon carcinoma spheroids placed in hand-made agarose beds in Ibidi µ-slides [17]. With this setup, we were able to show the magnetic accumulation of SPION^MTO^, and the consequent reduction of spheroid size. Due to the complicated production, poor reproduction, variation in the slides and laborious recovery of the spheroids for analysis, we aimed to establish a more standardized method. We modified the commercially available MIVO^®^ (“multi in vivo organ”) device to meet our needs. The system consisted of uniform tubes and chambers, which could be assembled together individually. This system had been used previously to investigate cisplatin efficacy towards 3D SKOV-3 cell-laden alginate hydrogels [35]. Instead of using transwells carrying the spheroids, we put the spheroids directly into the chambers. This was necessary, because the magnetic field was strongest at the bottom of the chambers (Figure 4B). To mimic the metabolism and elimination of the chemotherapeutics from the body, we changed one tube with the containing fluid three times, and replaced it with fresh medium. With this setup, it was possible to accumulate SPIONs together with MTO in the chambers, as detected by HPLC and AES, respectively (Figure 3D,E). The accumulation of MTO significantly inhibited the growth of the tumor spheroids (Figure 4C–E), and increased the amount of detached dead cells from the spheroids (Figure 4F,G), compared to the untreated controls, and compared also to free MTO, which represents the standard therapy.

Analogously, we used the system for investigating the magnetic enrichment of SPION-loaded cells, which are intended for use in targeted immunotherapy. Similarly to previous experiments, where we functionalized T cells, we were able to load PBMCs with Cit-SPIONs [20,21,23]. Using a peristaltic pump with a flow rate of 9.6 mL/min, we had already shown that it was possible to magnetically accumulate the SPION-loaded primary T cells at the desired place in Ibidi µ-slides, even when the flow had a higher velocity [20]. Others had loaded Jurkat cells as well as primary murine T cells with particles coated by 3-aminopropyl-triethoxysilane (APS) and had proofed their magnetic accumulation in a flow chamber assay [51]. Extending those previous setups, we additionally used spheroids to monitor the anti-tumor efficacy of stimulated PBMCs after their accumulation. We found that SPION-loaded PBMCs exhibited a stronger anti-tumor effect when magnetically enriched, as shown by the decreased size of the inner spheroid core, compared to the untreated controls and the non-loaded PBMCs (Figure 6).

Further tests will be conducted, to consider flow velocities, as the currently applied 1.08 mL/min is significantly slower than blood flow, which has a speed of 3–26 mL/min in arteries and 1.2–4.8 mL/min in veins, when taking into account the corresponding vessel diameters [52]. Presently, a faster circulation bears the risk of the spheroids being dragged out of the chamber into the tube system; embedding the spheroids in a matrix might help to fix them in the chambers. In addition, putting the spheroids in transwells into the chambers would enable us to build a more physiological environment, e.g., with the circulating media (“blood flow”) separated from the tumor by an endothelial layer, forming a biological barrier.

Stronger magnets and an improved magnetic field arrangement might also improve the accumulation of the SPIONs and SPION-loaded cells. As depicted, different magnets were used for the accumulation of the SPIONs and the enrichment of the Cit-SPION-loaded PBMCs. It was noticeable that the magnetic field for the magnetic cell targeting appeared in a more decentralized fashion (Figure 3B): however, we assume that here the flow was decelerated for the SPIONs or loaded cells, respectively, which would allow for sedimentation and therefore an enrichment resulting in a similar outcome, compared to the more centralized magnetic field (Figure 4B). For a better understanding of the system, simulations of the magnetic field must be combined with simulations of the flow. In addition, a variability in the distance of the magnet to the spheroid should be included, as not every tumor is reachable on the surface. In order to analyze the effects on peripheral healthy cells, various cell types could be distributed in chambers between the tubes, and analyzed afterwards. In the future, several parameters could be changed (type of tumor or chemotherapeutics), or variables could be added to the system (e.g., cytokines, endothelial cell layers, complex tissues), to create an even more realistic setup.

## 5. Conclusions

We established an in vitro system to analyze the magnetic accumulation of drug-loaded SPIONs or SPION-loaded cells and their effect on tumor spheroids. To the best of our knowledge, the presented system is the first non-handcrafted system to combine spheroids under dynamic flow with magnetic particles/cells and magnetic forces. Especially for nanomedical investigations employing magnetic transporters or cells, our application can bridge the gap between static setups and in vivo experiments.

## Figures and Tables

**Table 1:** Summary of the used concentrations.

Substrate	Concentration		
MTO	5 µM	0.5 µM	0.05 µM
SPION	225 µg Fe mL	22.5 µg Fe/mL	2.25 µg Fe/mL
SPION^MTO^	5 µM MTO	0.5 µM MTO	0.05 µM MTO
	+225 µg Fe/mL	+22.5 µg Fe/mL	+2.25 µg Fe/mL

MTO: mitoxantrone; SPION: superparamagnetic iron oxide nanoparticle; Fe: iron.

**Table 2:** Summary of the physicochemical parameters for the used SPION systems.

	Hydrodynamic Diameter (nm)	PDI (a.u.)	ζ-Potential at pH 7.3 (mV)	Vol. Suscepti- Bility × 10^−3^ (a.u.)
Cit-SPION	51 ± 1	0.208 ± 0.051	−44.6 ± 9.1	4.32 ± 0.18
Caf-SPION	63 ± 4	0.213 ± 0.014	−47.7 ± 6.5	3.36 ± 0.05
SPION	73 ± 2	0.225 ± 0.011	−33.9 ± 4.9	3.11 ± 0.12
SPION^MTO^	70 ± 1	0.193 ± 0.004	-	-

Z-Avg.: Z-average; PDI: polydispersity index; Vol.: volumetric; Cit: citrate; Caf: caffeic acid; BSA: bovine serum albumin; SPION: superparamagnetic iron oxide nanoparticles; MTO: mitoxantrone.

## Data Availability

The data presented in this study are available on request from the corresponding author.

## Appendix A

### Appendix A.1. Synthesis of CafPFP

Caffeic acid pentafluorophenyl ester (CafPFP) was synthesized, following the method of Williams et al. [36]. In short, 790 mg of caffeic acid was dissolved in 10 mL of DMF. While stirring with a magnetic stirrer, 570 µL of pyridine was added. Afterwards, 1.21 mL of pentafluorophenyl trifluoride was added, and the reaction was allowed to complete at room temperature for 2 h while mixing. Then, 30 mL of dichloromethane was added, and the reaction mixture was transferred to a separating funnel. The solution was washed 5× with 25 mL of 1 M HCl, by discarding the upper aqueous phase, and then dried over magnesium sulfate powder. The solution was filtered and concentrated using a rotary evaporator (Hei-Vap Precision, Heidolph, Germany). Using a chromatography column filled with silica gel, and a solvent ratio of petroleum ether/ethyl acetate 3/2, the crude product was purified and dried using the rotary evaporator. The gray-yellow CafPFP powder was stored dry and with light protection, until further use.

### Appendix A.2. Synthesis of Caf-SPION

Caf-stabilized SPIONs (Caf-SPION) were synthesized by adapting a protocol described by Mühlberger et al. [21]. A solution with an iron content of 13.2 mg/mL was generated by dissolving iron (II) and iron (III) salts in di. H_2_O. An aqueous solution of 25% NH_3_ was rapidly injected into the iron salt solution while vigorously stirring in an inert atmosphere. The solution turned black, due to the formation of a SPION dispersion. The dispersion was further mixed for 10 min at room temperature. A solution of 15 mL of pure ethanol containing 20 mg/mL CafPFP was injected into the dispersion, while stirring at 400 rpm. Immediately after the injection, the temperature of the reaction solution was raised to 90 °C, using a heating mantle. The reaction was allowed to continue for 30 min under reflux. After cooling to room temperature, the dispersion was transferred to falcon tubes for magnetic separation, using a strong magnet. The particles were washed twice, using 60 mL of pure ethanol. Afterwards, the particles were dried for 4 h with the help of a drying chamber. The dried particles were redispersed in 30 mL of di. H_2_O by ultrasound treatment for 3 min. To exclude large agglomerates, the Caf-SPIONs were filtered through a 0.2 µm filter membrane, and stored at 4 °C until further use. All particle batches were synthesized in triplicate (*n* = 3).

### Appendix A.3. Synthesis of Caf-BSA-SPIONs (SPION)

Albumin modification was achieved by slightly changing the protocol of Zaloga et al. [11]. Briefly, 38 mL of CafPFP-SPIONs, with a total iron content of 100 mg, were added to 10 mL of a 20 *w*/*v*% bovine serum albumin (BSA) solution, while stirring at 600 rpm, using an overhead stirrer. After 2 min of stirring, 2 mL of 0.1 M NaOH solution was added. The reaction solution was further stirred for 24 h at room temperature. Once the reaction was completed, 50 mL of di. H_2_O was added, to dilute the particle dispersion for washing by tangential flow filtration (KrosFlo Reseach II, Spectrum Labs, USA). The SPIONs were washed from the unbound albumin with 500 mL of di. H_2_O, using a 100 kDa hollow fiber filter module, and concentrated back to a total volume of 25 mL. The SPIONs were stored at 4 °C until further use.

**Table A1:** Values for the parameters of the simulation form Figure 2.

Parameter	Value in mm	Parameter	Value in mm
$D_{mag,1}$	23	$d_{mag,2,sim}=d_{mag,3,sim}$	3
$D_{mag,1.1}$	9.46	$D_{mag,3}$	32
$D_{mag,1.2}$	4.5	$D_{mag,3.1}$	11.7
$t_{1}$	2.48	$D_{mag,3.2}$	5.5
$h_{mag,1}$	4	$t_{2}$	3.1
$d_{mag,1,sim}$	2	$h_{mag,3}$	7
$D_{sim,1}$	16	$d_{mag,3,sim}$	3
$D_{sim,2}$	15	L	10
$D_{sim,3}$	8	d	1
$h_{sim,1}$	6	h	0.5
$h_{sim,2}$	2		
$h_{sim,3}$	2		
$D_{mag,2}$	15		
$h_{mag,2}$	8		

*D*: diameter, *t*: dip, *h*: height, *d*: distance, *L*: length.
